# Second Language Experience Facilitates Sentence Recognition in Temporally-Modulated Noise for Non-native Listeners

**DOI:** 10.3389/fpsyg.2021.631060

**Published:** 2021-04-07

**Authors:** Jingjing Guan, Xuetong Cao, Chang Liu

**Affiliations:** Department of Speech, Language, and Hearing Sciences, University of Texas at Austin, Austin, TX, United States

**Keywords:** sentence recognition in noise, temporally-modulated noise, non-native speakers, masking release from temporal modulation, second language experience

## Abstract

Non-native listeners deal with adverse listening conditions in their daily life much harder than native listeners. However, previous work in our laboratories found that native Chinese listeners with native English exposure may improve the use of temporal fluctuations of noise for English vowel identification. The purpose of this study was to investigate whether Chinese listeners can generalize the use of temporal cues for the English sentence recognition in noise. Institute of Electrical and Electronics Engineers (IEEE) sentence recognition in quiet condition, stationary noise, and temporally-modulated noise were measured for native American English listeners (EN), native Chinese listeners in the United States (CNU), and native Chinese listeners in China (CNC). Results showed that in general, EN listeners outperformed the two groups of CN listeners in quiet and noise, while CNU listeners had better scores of sentence recognition than CNC listeners. Moreover, the native English exposure helped CNU listeners use high-level linguistic cues more effectively and take more advantage of temporal fluctuations of noise to process English sentence in severely degraded listening conditions [i.e., the signal-to-noise ratio (SNR) of −12 dB] than CNC listeners. These results suggest a significant effect of language experience on the auditory processing of both speech and noise.

## Introduction

In daily life, speech sounds are usually perceived in noisy and complex listening environments. A number of studies have demonstrated that speech recognition in noise is much harder for non-native listeners than for native listeners regardless of stimuli are either vowels, consonants, words, or sentences (Mayo et al., 1997; Cooke et al., 2008; Cutler et al., 2008; Shi, 2010; Jin and Liu, 2012; Mi et al., 2013; Rimikis et al., 2013; Guan et al., 2015; Zinszer et al., 2019). Even though non-native listeners could achieve comparable performance with native listeners in quiet, non-native listeners performed much worse than native listeners in long-term speech-shaped noise (LTSSN) and multi-talker babble (MTB; Garcia Lecumberri and Cooke, 2006; Cutler et al., 2008). The difficulties of non-native listeners’ speech perception in noise were mainly impacted by their language backgrounds including their native language (L1) and second language (L2) experience, and listening conditions (Jin and Liu, 2012; Mi et al., 2013; Guan et al., 2015). For example, Jin and Liu (2012) reported that two groups of English non-native listeners (native Chinese and native Korean listeners) had similar scores in English sentence recognition in quiet and in LTSSN and these two non-native groups had significantly lower performance than native English listeners (EN). However, Korean listeners outperformed Chinese listeners in MTB, indicating that listeners’ native language (Korean or Chinese) might interfere with speech noise. However, in a follow-up study, Jin and Liu (2014) found no significant difference in English vowel identification in LTSSN and MTB, suggesting that L2 speech recognition in noises was dependent on L1 experience, the type of noise, and speech materials.

Compared with native listeners, non-native listeners with incomplete knowledge of target language and less automatic language processing had to spend more efforts to recognize speech in interfering noise (Kousaie et al., 2019). However, L2 exposure may help non-native listeners’ perception to be fine-tuned with sounds of L2. Previous studies showed two groups of native Chinese listeners, one in the US (CNU) with L2 exposure and the other in China (CNC) without L2 exposure, had similar performance of English vowel identification in quiet and LTSS noise, whereas CNU listeners showed significantly higher scores in MTB (Mi et al., 2013) and temporally modulated noise (Guan et al., 2015). Both studies argued that extensive native English (L2) experiences of CNU listener might help improve their capacity to perceive English vowels in temporally fluctuating maskers.

In most listening conditions, background noise is non-stationery, and amplitude of noise fluctuates over the time. It has been well-established that listeners with normal hearing including native and non-native listeners typically performed better in temporally modulated noise than in stationary noise (Festen and Plomp, 1990; Jin and Nelson, 2006; Broersma and Scharenborg, 2010; Stuart et al., 2010; Guan et al., 2015). This phenomenon, called masking release from the temporal modulation of noise, was usually explained as an ability to catch the glimpses of speech information during the short gaps of fluctuating noise and integrate these glimpses to restore the content of speech. Previous studies demonstrated that native listeners benefited more than non-native listeners from temporally fluctuating noise and obtained a larger masking release (Stuart et al., 2010; Guan et al., 2015). Guan et al. (2015) examined English vowel identification for three groups of listeners: native English (EN) listeners, CNU, and CNC listeners. As expected, EN listeners had significantly greater masking releases from temporal modulation in noise at low signal-to-noise ratios (SNRs) than the two groups of Chinese listeners. Moreover, CNU listeners had more masking releases for vowel identification than CNC listeners. Native English experiences for 1–3 years might help CNU listeners become better tuned to the temporal structure of English speech such that they had larger masking release. It was well demonstrated the basic psychophysical capacities to detect basic auditory tasks including temporal processing (Guan et al., 2015) were similar across listeners with different native language (L1) backgrounds and L2 exposure. Nevertheless, compared with basic auditory tasks, higher-level speech recognition in noise not only relied on listeners’ basic psychophysical capacities but also associated with listeners’ L1 and L2 experiences (Guan et al., 2015; Liu and Jin, 2015; Huo et al., 2016). Thus, this study was to investigate whether the CNU-CNC difference in using temporal dips of noise for English vowel identification could be extended in English sentence recognition, which was more ordinary in daily speech communication. Previous studies showed that speech materials significantly influenced the effect of L1 experience on L2 speech identification, i.e., significant difference in English sentence recognition in MTB between native Chinese and native Korean listeners (Jin and Liu, 2012), but no group difference for English vowel recognition in MTB between the two groups (Jin and Liu, 2014). Thus, the effect of L2 experiences (e.g., with or without L2 exposures) may also rely on speech materials (vowels and sentences).

## Materials And Methods

### Listeners

Three groups of listeners whose ages ranged from 20 to 35 years old were recruited based on their language experience: native EN, CNU, and CNC. The EN group comprised 10 listeners, while each of two native Chinese groups consisted of 14 listeners. All listeners had normal hearing with pure-tone thresholds ≤ 15 dB HL at octave intervals between 250 and 8,000 Hz (ANSI, 2010). Listeners in the EN and CNU groups were undergraduate or graduate students from the University of Texas at Austin, and listeners in the CNC group were undergraduate or graduate students from Beijing Normal University. Listeners in the two native Chinese groups started their formal school-based English education at 9–13 years old in China. The CNU group had internet-based Test of English as a Foreign Language (TOEFL) scores at least 80 with the United States residency of 1–3 years. Listeners in the CNC group passed the College English Test Band 4 (CET-4) in China without any residency history in English-speaking countries. The CET-4 is required for most of undergraduate students in China to receive their bachelor degree. All listeners received the consent form at the beginning of this study approved by the Ethics Review Board of Beijing Normal University and the Institutional Review Board of the University of Texas at Austin. They were paid for their participation.

### Stimuli

Sentence recognition performance was measured by using the Institute of Electrical and Electronics Engineers (IEEE) sentences, which was recorded from a female native English speaker. The IEEE sentences include 72 lists with each list composed of 10 sentences. Each sentence contains five key words. For a given listening condition, one sentence list was randomly selected, and was presented only once across all conditions.

Sentences were presented in either quiet or noise. Two types of noise, stationary LTSSN and temporally modulated noise, were presented at 70 dB SPL. The LTSSN was generated from Gaussian noise that was shaped by a filter with an average spectrum of a 12-talker babble (Kalikow et al., 1977). SNRs were manipulated at −12, −9, −6, −3, 0, and 3 dB. In addition, the temporally modulated noise was generated by applying a temporal-modulated envelope on the stationary LTSSN with the modulation frequency at 4, 16, and 64 Hz, and the modulation depth of 100%. The phase of the temporal modulation of noise was randomized between 0 and 2*π*. Speech and noise levels were calibrated with an AEC201-A IEC 60318-1 ear simulator by a Larson-Davis sound-level meter (Model 2800) with a linear weighting band.

### Procedure

For each listener, there were 25 experimental conditions (quiet, six SNRs × four modulation frequencies including the stationary noise). For a given condition, a randomly selected IEEE list was presented. Speech signals and noise, digitized at 12,207 Hz, were presented *via* SONY MDR-7506 headphones to the right ear of the listeners who were seated in a quiet test room. Stimulus presentation was controlled by the Tucker-Davis Technologies mobile processor (RM1). Listeners were seated in front of an LCD monitor and asked to type what they heard in a text box. After the sentence presentation, listeners were required to respond within 30 s. Sentence recognition score in percent correct for each sentence was evaluated offline and calculated according to the number of words correctly typed out of the five key words. The final sentence recognition performance for one listening condition was based on the average score of all 10 sentences. Before data collection, listeners were trained with a 5-min practice session of sentence recognition in a quiet listening condition to familiarize them with the experimental procedure.

After the practice session, listeners were examined with the test session in quiet first followed by the noise conditions. The order of the 24 noise conditions was randomized, which was manipulated by the software Sykofizx®.

## Results

### Sentence Recognition in Quiet

In quiet, the average sentence recognition score was 99.6% for the EN group, 70.7% for the CNU group, and 41.0% for the CNC group. In order to examine the effect of listener group on sentence recognition performance in quiet, a generalized linear model (GLM) was conducted with the scores in quiet as the dependent variable and listener group as a fixed effect. Results revealed that the main effect of listener group was significant [*χ*^2^(2) = 136.35, *p* < 0.001]. Pairwise comparisons demonstrated that the EN group performed significantly better than the two Chinese groups, while the CNU group significantly outperformed the CNC group (all *p* < 0.001).

### Sentence Recognition in Noise and Masking Release From Temporal Modulation

**Figure 1:**
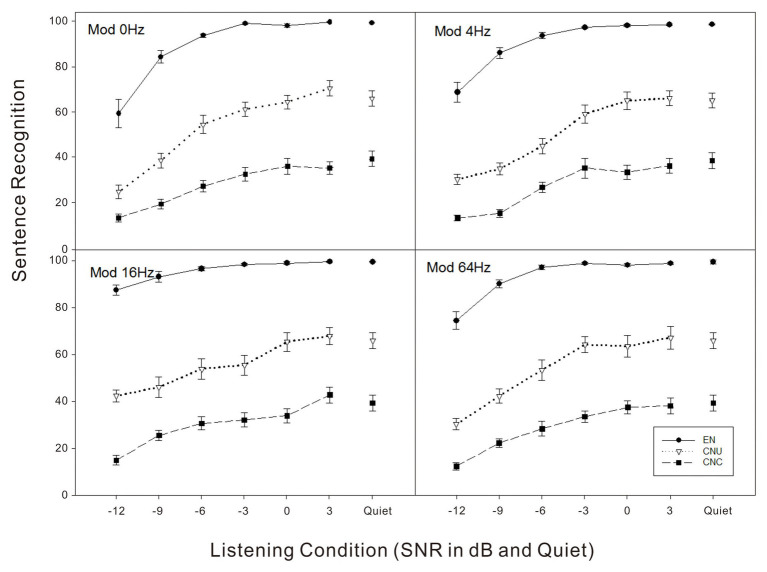
Sentence recognition scores in percent correct as a function of listening condition (quiet and signal-to-noise ratio in dB) for four noise conditions (top left - Mod 0 Hz: stationary noise; top right - Mod 4 Hz: noise temporally modulated at 4 Hz; bottom left - Mod 16 Hz: noise temporally modulated at 16 Hz; bottom right - Mod 64 Hz: noise temporally modulated at 64 Hz) for three groups of listeners: English-native (EN) listeners, Chinese-native listeners in the United States (CNU), and Chinese-native listeners in China (CNC).

As shown in Figure 1, sentence recognition scores in noise conditions were lower relative to scores in quiet for all the three groups. The native group outperformed the two non-native groups for all noise conditions. The data were analyzed using a generalized linear mixed model (GLMM) in SPSS software (version 26) where the performance of sentence recognition in noise as a dependent variable. In the model, fixed effects included listener group, SNR, modulation frequency, their two-way interactions, and the three-way interaction. To account for baseline differences in sentence recognition performance across subjects, we also included subject as a random effect.The GLMM analyses showed significant main effects of listener group, SNR, and modulation frequency (all *p* < 0.001). The two-way interactions between listener group and SNR [*F*(10,838) = 8.62, *p* < 0.001], SNR, and modulation frequency [*F*(15,838) = 4.67, *p* < 0.001], and the three-way interaction were significant [*F*(30,838) = 1.59, *p* < 0.05].

In order to further explore how the impact of listener group on sentence recognition in noise changed as the SNR varied, planned comparisons at each SNR indicated that native English listeners performed significantly better than two non-native Chinese groups regardless of modulation frequencies (all *p* < 0.05), while the CNU group obtained significantly higher scores of sentence recognition in noise than the CNC group across all the SNR conditions from −12 to +3 dB (all *p* < 0.05).

**Figure 2:**
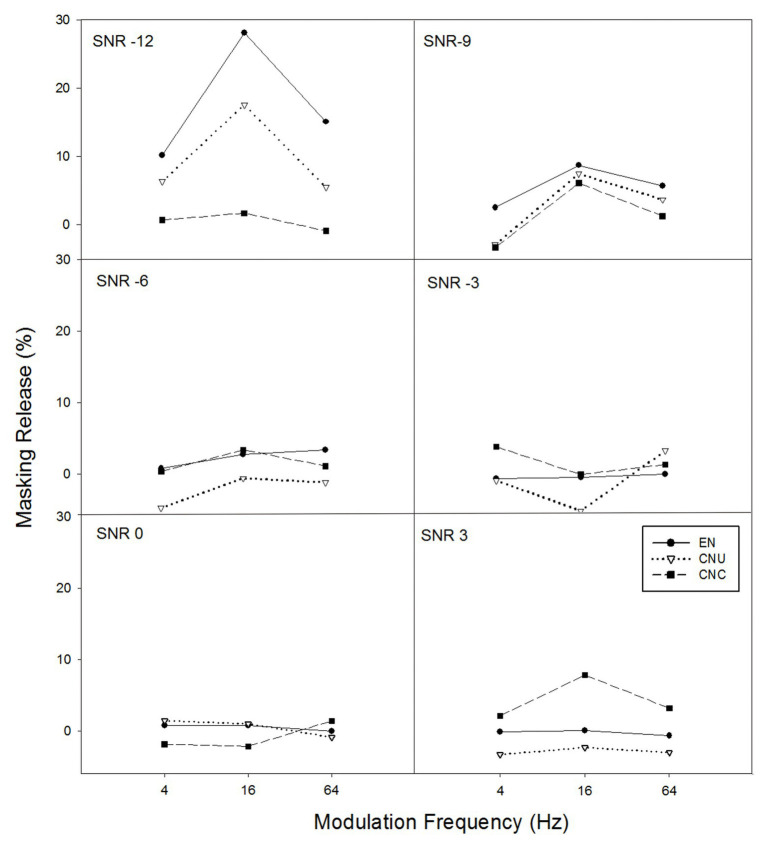
The masking release from the temporal modulation of noise in percent (e.g., scores in temporally-modulated noise minus scores in stationary noise) as a function of temporal modulation frequency of noise for six SNRs (−12, −9, −6, −3, 0, and 3 dB for each panel) for three groups of listeners: EN listeners, CNU, and CNC.

In order to examine the amount of masking release of sentence recognition from the temporal modulation of noise, the difference between the scores with temporally modulated noise (i.e., modulation frequency of 4, 16, and 64 Hz) and the scores with stationary noise were calculated (see Figure 2). With the masking release of sentence recognition in temporally modulated noise being a dependent variable, a GLMM model with fixed effects (i.e., listener group, SNR, modulation frequency, their two-way interactions, and the three-way interaction) were run. Subject was entered as a random effect factor. The GLMM model yielded significant main effects of modulation frequency [*F*(2,628) = 6.46, *p* < 0.05] and SNR [*F*(5,628) = 13.47, *p* < 0.001], a significant interaction between listener group and SNR [*F*(10,628) = 5.18, *p* < 0.001], and a significant interaction between SNR and modulation frequency [*F*(10,628) = 2.40, *p* < 0.05]. Follow-up analysis showed a significant effect of listener group only at the SNR of −12 dB [*F*(2,628) = 17.65, *p* < 0.001], indicating the EN group received significantly greater masking release than the CNU group whose masking release was significantly larger than that of the CNC group only at the SNR of −12 dB. No significant group differences were observed at other SNRs (all *p* > 0.05).

### Normalized Sentence Recognition Performance in Noise and Related Masking Release

**Figure 3:**
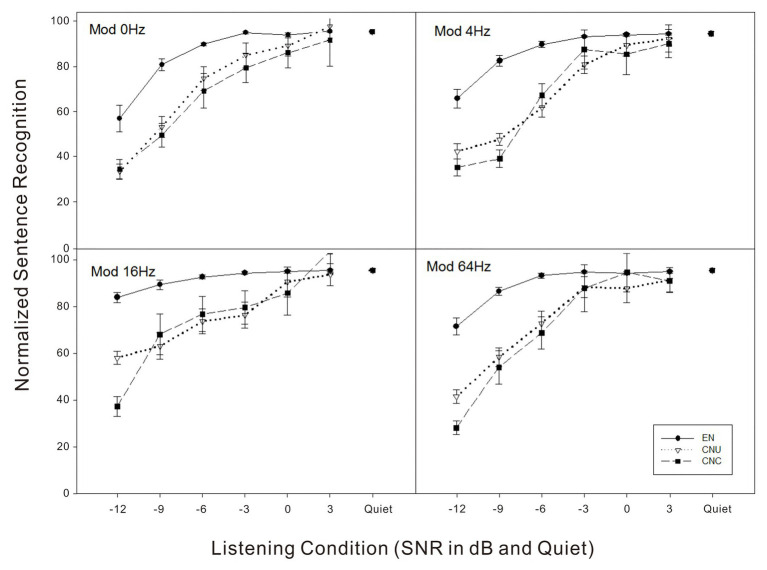
Normalized sentence recognition scores (e.g., recognition scores in noise/quiet divided by scores in quiet) as a function of listening condition (quiet and signal-to-noise ratio in dB) for four noise conditions (top left – Mod 0 Hz: stationary noise; top right – Mod 4 Hz: noise temporally modulated at 4 Hz; bottom left – Mod 16 Hz: noise temporally modulated at 16 Hz; and bottom right – Mod 64 Hz: noise temporally modulated at 64 Hz) for three groups of listeners: EN listeners, CNU, and CNC.

In order to rule out the effect of the language proficiency across the three listener groups, normalized scores of sentence recognition were computed by the percentage scores in noise listening conditions divided by the percentage scores in quiet for each listener. As shown in Figure 3, for example, the EN, CNU, and CNC groups had 84.4, 38.6, and 19.4% scores for sentence recognition in the stationary noise at the SNR of −9 dB. As the baseline, the scores of sentence recognition in quiet for the three groups were 99.6, 70.7, and 41.0%, respectively. Therefore, the normalized scores of sentence recognition were then 0.85 (84.4/99.6%), 0.55 (38.6/70.7%), and 0.47 (19.4/41.0%) for the EN, CNU, and CNC groups, respectively. The GLMM model on normalized sentence recognition performance revealed main effects of listener group, SNR, and modulation frequency were significant (all *p* < 0.05). The interaction between listener group and SNR [*F*(10,838) = 10.33, *p* < 0.001] and the interaction between SNR and modulation [*F*(15,838) = 2.64, *p* < 0.05] were also significant. *Post hoc* analysis showed that normalized performance of EN group was significantly better than the two non-native Chinese groups (all *p* < 0.05) while two Chinese groups had no significant difference at the SNRs of −12, −9, and −6 dB. No other significant differences among three listener groups were found at the SNRs of −3, 0, and 3 dB.

**Figure 4:**
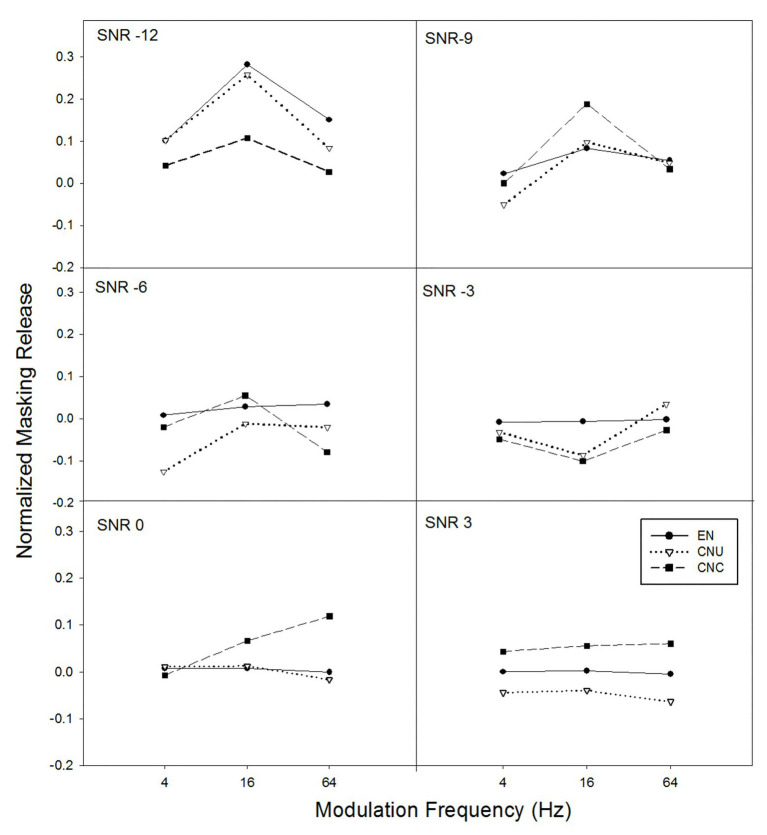
The normalized masking release from the temporal modulation of noise (e.g., normalized scores in temporally-modulated noise minus normalized scores in stationary noise) as a function of temporal modulation frequency of noise for six SNRs (−12, −9, −6, −3, 0, and 3 dB for each panel) for three groups of listeners: EN listeners, CNU, and CNC.

The amount of masking release from the temporal modulation of noise for normalized sentence recognition scores was also calculated by subtracting the normalized scores with stationary noise from the normalized scores with temporally modulated noise (i.e., modulation frequency of 4, 16, and 64 Hz; see Figure 4). The GLMM model was applied to examine the effects of listener group, SNR, modulation frequency, and their interactions on masking release for normalized sentence recognition performance. Results demonstrated that normalized masking release was significantly contributed by the main effects of modulation frequency [*F*(2,628) = 4.17, *p* < 0.05] and SNR [*F*(5,628) = 4.46, *p* < 0.05], and the interaction of listener group and SNR [*F*(10,628) = 2.10, *p* < 0.05] but not by the other main effect and interaction effects (all *p* > 0.05). *Post hoc* analysis suggested listeners achieved significantly greater normalized masking release at the modulation frequency of 16 Hz than at 4 Hz, while no significant differences were observed for other comparisons among modulation frequencies. Furthermore, a significant group difference on normalized masking release was only found at the SNR of −12 dB [*F*(2,628) = 5.85, *p* < 0.05] but not at other SNR conditions. Specifically, as shown in Figure 4, normalized masking release in severely degraded listening conditions (i.e., the SNR of −12 dB) for the EN and CNU groups had significantly greater masking release than the CNC group (both *p* < 0.05), while the EN and CNU achieved similar masking release (*p* > 0.05).

### Correlation Between Sentence Recognition Performance and Masking Release

In order to examine whether English proficiency level of Chinese-native speakers significantly affected the masking release from the temporal modulation of noise, the correlation between sentence recognition scores in quiet and the masking release at each listening condition (e.g., at a given SNR and one temporal modulation frequency of noise) was analyzed. Results indicated a significant correlation for Chinese-native speakers including both CNU and CNC listeners at the SNR of −12 dB for the temporal modulation frequency of 16 Hz for both original (Pearson *r* = 0.546, *p* < 0.05) and normalized (Pearson *r* = 0.392, *p* < 0.05) data. That is, for Chinese-native speakers, better sentence recognition in quiet and larger masking release from the temporal modulation in noise.

## Discussion

### Disadvantage for Non-native Listeners on Sentence Recognition in Noise

Consistent with previous studies (Mayo et al., 1997; Shi, 2010; Jin and Liu, 2012; Zhong et al., 2019), EN listeners’ performance in quiet (99.6%) showed remarkable native language advantages over the two non-native groups (CNU: 70.7%; CNC: 41.0%). However, CNU listeners with native English experiences of 1–3 years significantly outperformed CNC listeners in sentence recognition in quiet, in contrast with the findings of our previous studies (Mi et al., 2013; Guan et al., 2015), which reported there was no difference in English vowel identification in quiet between CNU (accuracy: 70%) and CNC listeners (accuracy: 64%). It was possibly because that sentences contain redundant cues (e.g., acoustic-phonetic, semantic, syntactic, and prosodic cues), while vowels have limited acoustic-phonetic cues. Compared with vowels, sentences were more related with language experience, especially when the IEEE sentences were contextually more complicated than other sentence materials (Nilsson et al., 1994). Zhong et al. (2019) found that for English sentence recognition in quiet and four-talker babble, EN listeners weighted speech cues including semantic and syntactic cues equally, whereas CNU listeners primarily relied on semantic information. That is, the gap between EN and CNU listeners was larger as the sentences became from high to low predictability. The findings of this study combined with those of previous studies in our laboratories suggested that the non-native disadvantage in English sentence perception was significantly dependent on the complexity of sentences, e.g., for sentences with high predictability, there was only 5–10% gap between EN and CNU listeners (Jin and Liu, 2012; Zhong et al., 2019), while for sentences in low predictability, the gap was enlarged to more than 20% in this study and study of Zhong et al.(2019). Altogether, these results indicate that non-native listeners have significant challenges of processing L2 speech signals when primarily using a bottom-up approach, i.e., semantic information as a top-down cue was missing in low-predictability sentences.

Moreover, the comparison between CNU and CNC listeners indicated no difference in vowel identification (Guan et al., 2015), but a significant difference in sentence recognition in this study suggest that native English experience of 1–3 years may improve non-native listeners’ processing of high-level linguistic cues of English speech sounds, but not the processing of phonetic cues, the improvement of which may need specific phonetic training.

In this study, the two non-native groups started to learn English around 9–13 years old, who were later learners of the second language. The non-native disadvantage is consistent with previous findings that late bilingual speakers (e.g., age of acquisition about 10–13 years old) were not able to recognize sentences in noise as efficiently as monolingual English speakers (Mayo et al., 1997; Shi, 2010). Further study with more participant variables, such as L2 learning environment, motivation, L2 learning styles, and cognitive abilities, needs to be included and investigated in the future, which may also contribute to the challenges of perceiving L2 speech in non-optimal listening conditions for nonnative listeners (Yang et al., 2017; Kousaie et al., 2019). In order to further discuss sentence recognition in noise by minimizing the effect of English proficiency for Chinese-native listeners, normalized scores were calculated, and the noise effect was then analyzed based on the normalized scores (see Figure 3). Results indicated that only in severely degraded listening conditions (i.e., SNRs of −12 to −6 dB,), the native English group had significantly better scores than the two non-native groups. There was no significant group difference for normalized sentence recognition scores at more favorable listening conditions (SNRs from −3 to +3 dB). Results suggested that when the language proficiency was controlled (e.g., the sentence recognition scores were normalized), noise seemed to affect the three groups of listeners similarly at medium and high SNRs, but influenced non-native listeners more negatively than native listeners at low SNRs.

In this study, in acoustically degraded conditions, non-native listeners showed disadvantages in sentence recognition in noise from the SNR of −12 to +3 dB. It should be noted that whether background noise causes greater difficulty for non-native listeners than for native listeners depends on a variety of factors, such as SNR, speech materials, and noise type. For example, for English vowel identification, LTSSN did not enlarge the gap between EN and Chinese-native listeners, while MTB did for high and middle SNRs (Mi et al., 2013). On the other hand, MTB made no changes in the non-native disadvantages in quiet for CNU listeners for broad SNRs, but resulted in larger non-native advantage for CNC listeners at very low SNRs (e.g., −15 and −20 dB SNR). For sentence recognition, the gap between native and non-native listeners became larger for sentence recognition in MTBs for CNU listeners than in quiet (Jin and Liu, 2012; Zhong et al., 2019), particularly at high and medium SNRs. Thus, the effect of SNRs on non-native disadvantage in speech recognition is quite complicated, depending on a number of factors, such as speech materials, noise type, listeners’ L2 experience, and SNR.

### Masking Release From Temporal Modulation for Non-native Listeners Mediated With Language Experience

As expected, both native and non-native listeners benefited from the temporal modulation of noise, especially at the low SNRs (e.g., −12 dB; see Figure 2). With regard to the masking release from temporal modulation of noise across the three groups, EN and CNU listeners obtained larger masking releases than CNC listeners at the SNR of −12 dB, consistent with previous findings in English vowel identification (Guan et al., 2015) and Chinese vowel identification (Li et al., 2016). Such group difference in the masking release at low SNRs was also observed in Figure 4 when the effect of English proficiency was minimized (e.g., the scores of sentence recognition were normalized). Thus, it is likely that as for either vowel or sentence testing, smaller benefit from the temporal modulation of noise for CNC listeners may be due to the lack of native English experience with the temporal structure of the target language.

The amount of masking release from temporal modulation at the low SNRs for native and nonnative listeners depended on the temporal modulation frequency of background noise. It was found that in this study, the masking release obtained by listeners in both Figures 2, 4 was the significantly highest at the modulation frequency of 16 Hz, which was consistent with previous studies (Guan et al., 2015; Li et al., 2016). Compared with performance of speech perception in temporally-modulated noise at either slower (e.g., 4 Hz) or faster modulation frequency (e.g., 64 Hz), higher scores were found at the modulation frequency between 10 and 20 Hz (Gustafsson and Arlinger, 1994). The less masking release at the modulation frequency of 4 Hz was likely due to phonemes, and syllables were more easily interfered in background noise with similar temporal characteristics. Moreover, 64-Hz modulation dips might be too short for auditory responses to recover so as to aid speech recognition (Wojtczak et al., 2011).

Altogether, the previous studies (Stuart et al., 2010; Mi et al., 2013; Guan et al., 2015; Li et al., 2016) including the current one suggest a significant difference in the effectiveness of using temporal dips in noise for speech perception between native English listeners and native Chinese listeners, and between native Chinese listeners with different English experiences (e.g., CNU and CNC listeners). However, it should be also noted that Zhang et al. (2011) reported that there was no difference in the temporal dip listening for English word recognition between EN and CNU listeners. The discrepancy in the language experience effect on temporal dip listening could be due to the difference in speech materials (e.g., vowels in Guan et al., 2015, sentences in this study and Stuart et al., 2010, and words in Zhang et al., 2011) and temporal features of background noise (e.g., temporally modulated noise at 4, 16, and 64 Hz in Guan et al., 2015 and this study, and interrupted noise in Stuart et al., 2010 and Zhang et al., 2011). Another possibility is the different SNRs used across these studies. As suggested in study of Guan et al. (2015) and this study, the group difference in temporal dip listening was found only at quite negative SNRs (e.g., −12 dB), while the SNR of study of Zhang et al. (2011) was at −10 dB. Thus, more systematical studies are needed to fully examine the language experience impact on listening in temporal glimpses with a focus on the two factors: speech materials and acoustic features of noise.

The group difference in temporal dip listening in this and previous studies mentioned above may be due to the difference in the temporal structures of speech between English and Mandarin Chinese, i.e., English speech is more temporally dynamic than Mandarin Chinese (Calandruccio et al., 2010). Regular exposures to English speech environment seem to benefit native Chinese listeners by taking better advantage in using temporal glimpses of noise for speech recognition. These results suggest that speech training in temporally-varying noise may be needed and beneficial for non-native listeners if one’s study is to improve their speech perception in dynamic noise backgrounds.

### Limitation and Future Directions

One limitation of the current study was several variables of Chinese-native speakers were not well controlled, particularly English proficiency. As indicated above, the sentence recognition scores in quiet were significantly correlated with the masking release from the temporal modulation of noise at the SNR of −12 dB and modulation frequency of 16 Hz for Chinese-native listeners. This implies that Chinese-native listeners’ English proficiency may play an import role in temporal dip listening of English speech perception. Thus, a standardized and comprehensive speech perception test including the perception of English phonemes, words, and sentences is needed in future studies to quantify English proficiency level.

Another limitation was that several variables of L2 learners were not well manipulated, such as listeners’ cognitive function (e.g., working memory), learning style, L2 learning duration, and perceptual weights on speech redundant cues. Systematic studies are needed to examine the effects of these factors on L2 listeners’ English speech recognition in quiet and noisy listening conditions, especially in temporally-fluctuating noise.

## Conclusion

English listeners generally recognized sentences in quiet and noise more accurately than native Chinese listeners, while CNU listeners had better scores of sentence recognition than CNC listeners. The native English experience helped CNU listeners process high-level linguistic cues more effectively and take greater advantage of the temporal fluctuations of noise in severely degraded listening conditions (i.e., SNR of −12 dB) compared to their counterparts in China (CNC).

## Data Availability Statement

The raw data supporting the conclusions of this article will be made available by the authors, without undue reservation.

## Ethics Statement

The studies involving human participants were reviewed and approved by the Ethics Review Board of Beijing Normal University and the Institutional Review Board of the University of Texas at Austin. The patients/participants provided their written informed consent to participate in this study.

## Author Contributions

JG contributed to experimental design, data collection, data analysis, and manuscript writing. XC contributed to experimental design, data analysis, and manuscript writing, while CL made contributions to experimental design, data analysis, and manuscript writing. All authors contributed to the article and approved the submitted version.

### Conflict of Interest

The authors declare that the research was conducted in the absence of any commercial or financial relationships that could be construed as a potential conflict of interest.

## References

[ref1] ANSI (2010). S3.6, Specification for Audiometers. (New York: Acoustical Society of America)

[ref2] Broersma M. Scharenborg O. (2010). Native and non-native listeners’ perception of English consonants in different types of noise. Speech Comm. 52, 980–995. 10.1016/j.specom.2010.08.010

[ref3] Calandruccio L. Dhar S. Bradlow A. R. (2010). Speech-on-speech masking with variable access to the linguistic content of the masker speech. J. Acoust. Soc. Am. 128, 860–869. 10.1121/1.3458857, PMID: 20707455 PMC2933260

[ref4] Cooke M. Garcia Lecumberri M. L. Barker J. (2008). The foreign language cocktail party problem: energetic and informational masking effects in non-native speech perception. J. Acoust. Soc. Am. 123, 414–427. 10.1121/1.2804952, PMID: 18177170

[ref5] Cutler A. Garcia Lecumberri M. L. Cooke M. (2008). Consonant identification in noise by native and non-native listeners: effects of local context. J. Acoust. Soc. Am. 124, 1264–1268. 10.1121/1.2946707, PMID: 18681612

[ref6] Festen J. Plomp R. (1990). Effects of fluctuating noise and interfering speech on the speech reception threshold for impaired and normal hearing. J. Acoust. Soc. Am. 88, 1725–1736. 10.1121/1.400247, PMID: 2262629

[ref7] Garcia Lecumberri M. Cooke M. (2006). Effect of masker type on native and non-native consonant perception in noise. J. Acoust. Soc. Am. 119, 2445–2454. 10.1121/1.2180210, PMID: 16642857

[ref8] Guan J. Liu C. Tao S. Mi L. Wang W. Dong Q. (2015). Vowel identification in temporal-modulated noise for native and non-native listeners: effect of language experience. J. Acoust. Soc. Am. 138, 1670–1677. 10.1121/1.4929739, PMID: 26428804

[ref9] Gustafsson H. A. Arlinger S. D. (1994). Masking of speech by amplitude-modulated noise. J. Acoust. Soc. Am. 95, 518–529. 10.1121/1.408346, PMID: 8120263

[ref10] Huo S. Tao S. Wang W. Li M. Dong Q. Liu C. (2016). Auditory detection of non-speech and speech stimuli in noise: native speech advantage. J. Acoust. Soc. Am. 139, EL161–EL166. 10.1121/1.4951705, PMID: 27250202

[ref11] Jin S.-H. Liu C. (2012). English sentence recognition in speech-shaped noise and multi- talker babble for English-, Chinese-, and Korean-native listeners. J. Acoust. Soc. Am. 132, EL391–EL397. 10.1121/1.4757730, PMID: 23145700

[ref12] Jin S.-H. Liu C. (2014). English vowel identification in quiet and noise: effects of listeners’ native language background. Front. Neurosci. 8:305. 10.3389/fnins.2014.00305, PMID: 25400538 PMC4212258

[ref13] Jin S.-H. Nelson P. (2006). Speech perception in gated noise: the effects of temporal resolution. J. Acoust. Soc. Am. 119, 3097–3108. 10.1121/1.2188688, PMID: 16708964

[ref14] Kalikow D. N. Stevens K. M. Elliott L. L. (1977). Development of a test of speech intelligibility in noise using sentence materials with controlled word predictability. J. Acoust. Soc. Am. 61, 1337–1351. 10.1121/1.381436, PMID: 881487

[ref15] Kousaie S. Baum S. Phillips N. A. Gracco V. Titone D. Chen J. K.. (2019). Language learning experience and mastering the challenges of perceiving speech in noise. Brain Lang. 196:104645. 10.1016/j.bandl.2019.104645, PMID: 31284145

[ref16] Li M. Wang W. Tao S. Dong Q. Guan J. Liu C. (2016). Mandarin Chinese vowel- plus-tone identification in noise: effect of language experience. Hear. Res. 331, 109–118. 10.1016/j.heares.2015.11.007, PMID: 26611794

[ref17] Liu C. Jin S. H. (2015). Auditory detection of non-speech and speech stimuli in noise: effects of listeners’ native language background. J. Acoust. Soc. Am. 138, 2782–2790. 10.1121/1.4934252, PMID: 26627754

[ref18] Mayo L. H. Florentine M. Buus S. (1997). Age of second-language acquisition and perception of speech in noise. J. Speech Lang. Hear. Res. 40, 686–693. 10.1044/jslhr.4003.686, PMID: 9210123

[ref19] Mi L. Tao S. Wang W. Dong Q. Jin S.-H. Liu C. (2013). English vowel identification in long-term speech-shaped noise and multi-talker babble for English and Chinese listeners. J. Acoust. Soc. Am. 133, EL391–EL397. 10.1121/1.4800191, PMID: 23656099

[ref20] Nilsson M. Soli S. D. Sullivan J. A. (1994). Development of the hearing in noise test for the measurement of speech reception thresholds in quiet and in noise. J. Acoust. Soc. Am. 95, 1085–1099. 10.1121/1.408469, PMID: 8132902

[ref21] Rimikis S. Smiljanic R. Calandruccioa L. (2013). Nonnative English speaker performance on the basic english lexicon (BEL) sentences. J. Speech Lang. Hear. Res. 56, 792–804. 10.1044/1092-4388(2012/12-0178), PMID: 23275396

[ref22] Shi L.-F. (2010). Perception of acoustically degraded sentences in bilingual listeners who differ in age of English acquisition. J. Speech Lang. Hear. Res. 53, 821–835. 10.1044/1092-4388(2010/09-0081), PMID: 20220026

[ref23] Stuart A. Zhang J. Swink S. (2010). Reception thresholds for sentence in quiet and noise for monolingual English and bilingual mandarin-English listeners. J. Am. Acad. Audiol. 21, 239–248. 10.3766/jaaa.21.4.3, PMID: 20388450

[ref25] Wojtczak M. Nelson P. C. Viemeister N. F. Carney L. H. (2011). Forward masking in the amplitude-modulation domain for tone carriers: psychophysical results and physiological correlates. J. Assoc. Res. Otolaryngol. 12, 361–373. 10.1007/s10162-010-0251-2, PMID: 21181225 PMC3085689

[ref26] Yang X. Jiang M. Zhao Y. (2017). Effects of noise on English listening comprehension among Chinese college students with different learning styles. Front. Psychol. 8:1764. 10.3389/fpsyg.2017.01764, PMID: 29085317 PMC5650695

[ref100] Zhang J. Stuart A. Swink S. (2011). Word recognition in continuous and interrupted noise by monolingual and bilingual speakers. Can. J. Speech-Lang. Pathol. Audiol. 35, 322–331. PMID: 31046332

[ref27] Zhong L. Liu C. Tao S. (2019). Sentence recognition for native and non-native English listeners in quiet and babble: effects of contextual cues. J. Acoust. Soc. Am. 145, EL297–EL302. 10.1121/1.5097734, PMID: 31046332

[ref28] Zinszer B. D. Riggs M. Reetzke R. Chandrasekaran B. (2019). Error patterns of native and non-native listeners’ perception of speech in noise. J. Acoust. Soc. Am. 145, EL129–EL135. 10.1121/1.5087271, PMID: 30823795 PMC6365288

